# Bulbar urethrocavernous fistula in setting of inflatable penile prosthesis: a case report

**DOI:** 10.1186/s12894-021-00793-9

**Published:** 2021-02-13

**Authors:** Hannah Botkin, Brian Barnes, Amy Pearlman

**Affiliations:** University of Iowa Hospitals and Clinics, 200 Hawkins Drive, Iowa City, IA 52242 USA

**Keywords:** Urethrocavernous fistula, Inflatable penile prosthesis, Urethroplasty

## Abstract

**Background:**

Urethral injury or erosion of an inflatable penile prosthetic (IPP) cylinder is a rare complication of IPP placement. It can present with varying symptoms and management can be difficult with risk for future complications.

We present a patient with an eroded IPP who developed a secondary contralateral urethrocavernous fistula requiring repeat urethroplasty. We also describe the literature surrounding these complications and strategies to prevent them.

**Case presentation:**

A 69-year-old man with poorly controlled diabetes presented to our clinic with 6 months of intermittent white urethral discharge first noted after IPP removal and replacement by an outside urologist for device malfunction. Office cystoscopy revealed an eroded right-sided prosthetic cylinder in the bulbar urethra. The patient was taken to the operating room for IPP explantation with closure of right corporal defect, left sided malleable prosthesis placement, and primary excision with anastomosis of his bulbar urethra. A catheter was left in place for two weeks postoperatively, at which time a peri-catheter retrograde urethrogram was performed which showed no evidence of contrast extravasation and his catheter was subsequently removed. Several months later, he presented with recurrent urethral discharge without evidence of recurrent erosion on cystoscopy with development of scrotal abscesses following office cystoscopy, concerning for an unidentified urethral defect. He returned to the operating room for scrotal exploration and was noted on cystoscopy to have a pinpoint fistula between his left corporal body and his bulbar urethra. He underwent left sided malleable prosthetic explant, and non-transecting bulbar urethroplasty. Peri-catheter retrograde urethrogram two weeks later showed no contrast extravasation and he has had no recurrence of urethral discharge or scrotal abscesses since.

**Conclusions:**

Urethral erosion and urethrocavernous fistula formation are rare complications of penile prosthesis placement. Risks are elevated in patients with corporal fibrosis, diabetes, those undergoing penile implant revision surgery, and those requiring prolonged urethral catheterization.

## Background

Surgical placement of inflatable (IPP) or malleable penile prostheses (MPP) continue to be a viable option for the definitive management of erectile dysfunction (ED). The long-term reliability, as well as patient satisfaction, of these devices is generally high [1], but are not without risk of intraoperative or postoperative complication. Intraoperatively, the urethra may be injured at time of corporotomy or corporal dilation [2]. Post-operatively, risks include infection, device malfunction, and/or erosion. Risk of surgical complications are elevated in those with prior procedures, diabetes, smoking, and/or prior radiation [3].

Urethrocavernous fistula has been reported in patients with a history of proximal corporospongiosal shunt for priapism [4], injury during sexual activity [5], blunt penile trauma [6], or even spontaneously presenting with urethral bleeding [7], but has most commonly been reported after penile prosthetic surgery [3].

Here, we present a patient who experienced a rare complication of urethrocavernous fistula involving his bulbar urethra after prior IPP revision surgery.

## Case presentation

**Fig. 1:**
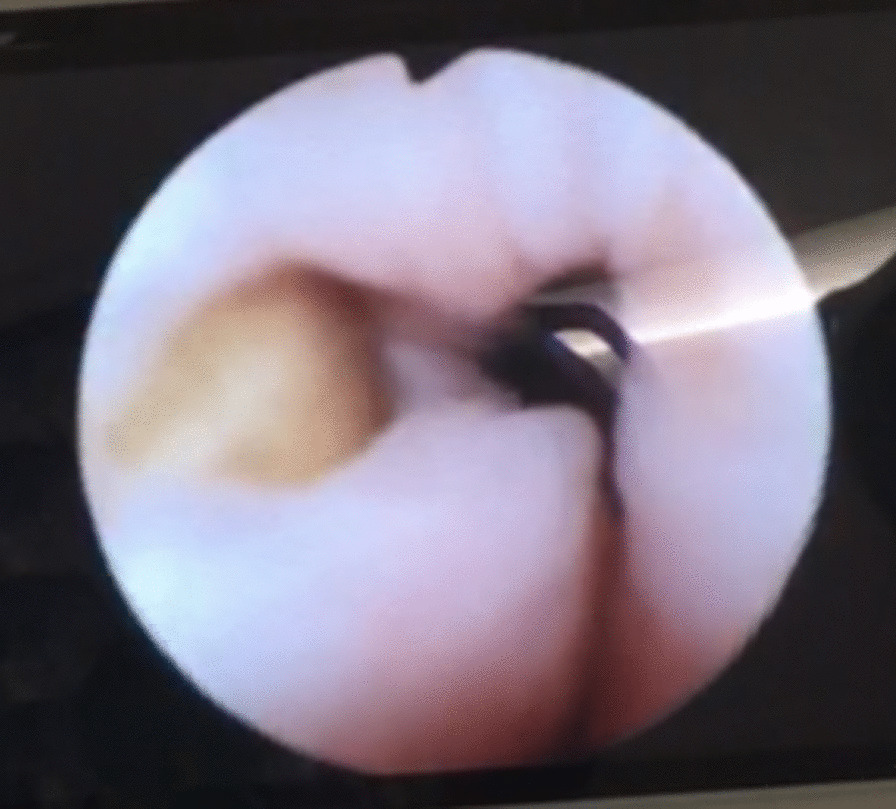
Urethrocavernous fistula as seen via cystoscopy (inflatable penile implant cylinder seen eroding into bulbar urethra at time of initial surgery with our team)

A 69-year-old African American male with hypertension, hyperlipidemia, chronic obstructive pulmonary disease, type 2 diabetes (hemoglobin A1C 8.4 %) presented to our clinic with approximately six months of intermittent white urethral discharge which began shortly after penoscrotal IPP removal and replacement by an outside urologist for prior IPP malfunction. No operative note was available for review. The discharge did not change in quantity after ejaculation or intercourse and was not associated with any urinary symptoms. Testing for sexually transmitted diseases by local urologist was negative. Clinic cystoscopy revealed an eroded right sided IPP cylinder involving the bulbar urethra (Fig. 1).

**Fig. 2:**
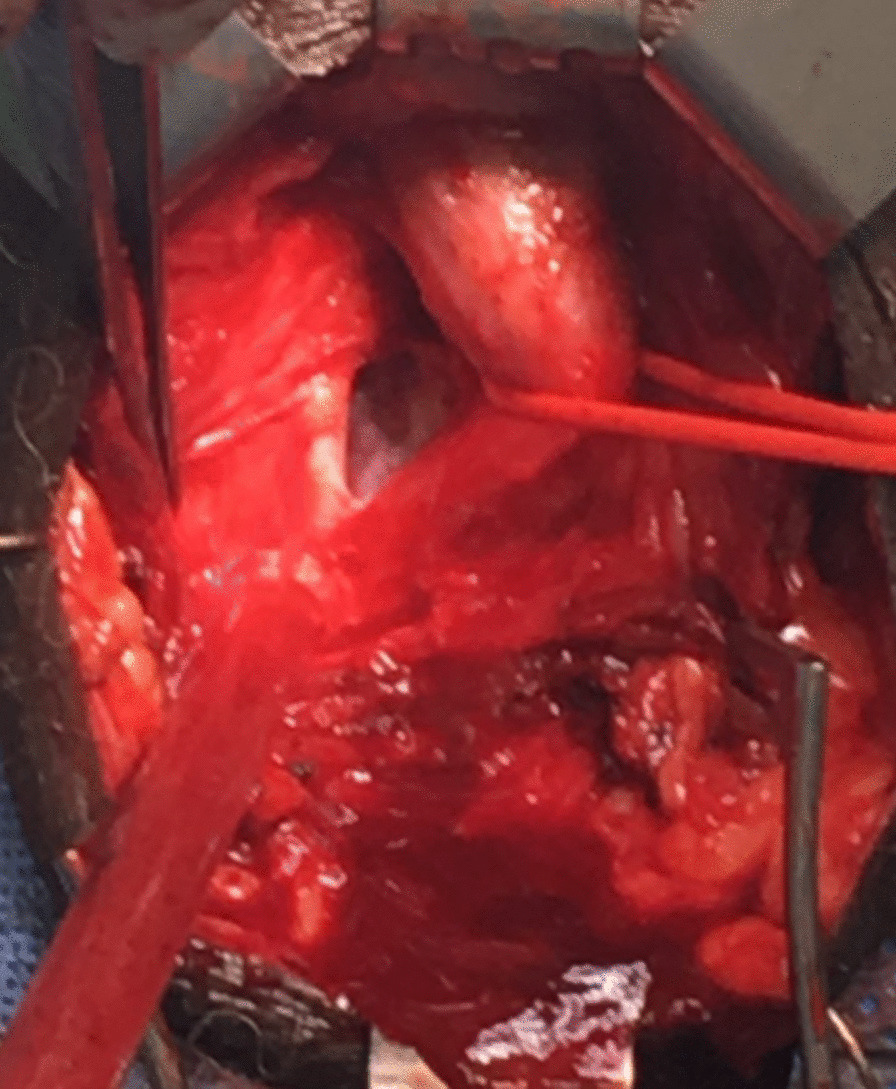
Urethrocavernous fistula within perineal incision after removal of right inflatable penile implant cylinder at time of initial surgery with our team

The patient was taken to the operating room for removal of all IPP components through a penoscrotal incision. Erosion of the right IPP cylinder was confirmed by visualizing a 0.5 cm defect into the right lateral aspect of the bulbar urethra (Fig. 2). A transecting excision and primary anastomosis (EPA) urethroplasty was then performed in the area of urethral erosion through a perineal incision. The corporal defect was closed separately. A14 French foley catheter was left in place with plans to remain in place for two weeks postoperatively. A 21 cm malleable prosthesis was placed in the left corpora (purposely undersized by 1 cm to avoid distal erosion).

A peri-catheter retrograde urethrogram (RUG) was performed 2 weeks postoperatively, which showed no evidence of contrast extravasation, and his catheter was removed. Six months postoperatively, he returned to clinic with continued urethral discharge and pain at the tip of his penis. Physical exam was concerning for impending MPP distal erosion. Office cystoscopy at that time showed a well-healed urethral anastomosis without visualization of the unilateral MPP cylinder, recurrent urethral erosion, or fistula. It was recommended that he undergo MPP explant (given concerns for impending distal erosion) but his surgery was delayed due to the COVID-19 pandemic in the context of his medical comorbidities.

Two weeks following office cystoscopy, the patient returned to clinic with new scrotal drainage and pain. Exam revealed an area of fluctuance concerning for scrotal abscess for which he underwent incision and drainage in clinic with evacuation of purulent material and clear fluid. He was prescribed antibiotics and scheduled for scrotal incision and drainage, MPP explant, and possible urethroplasty for presumed urethrocavernous fistula.

**Fig. 3:**
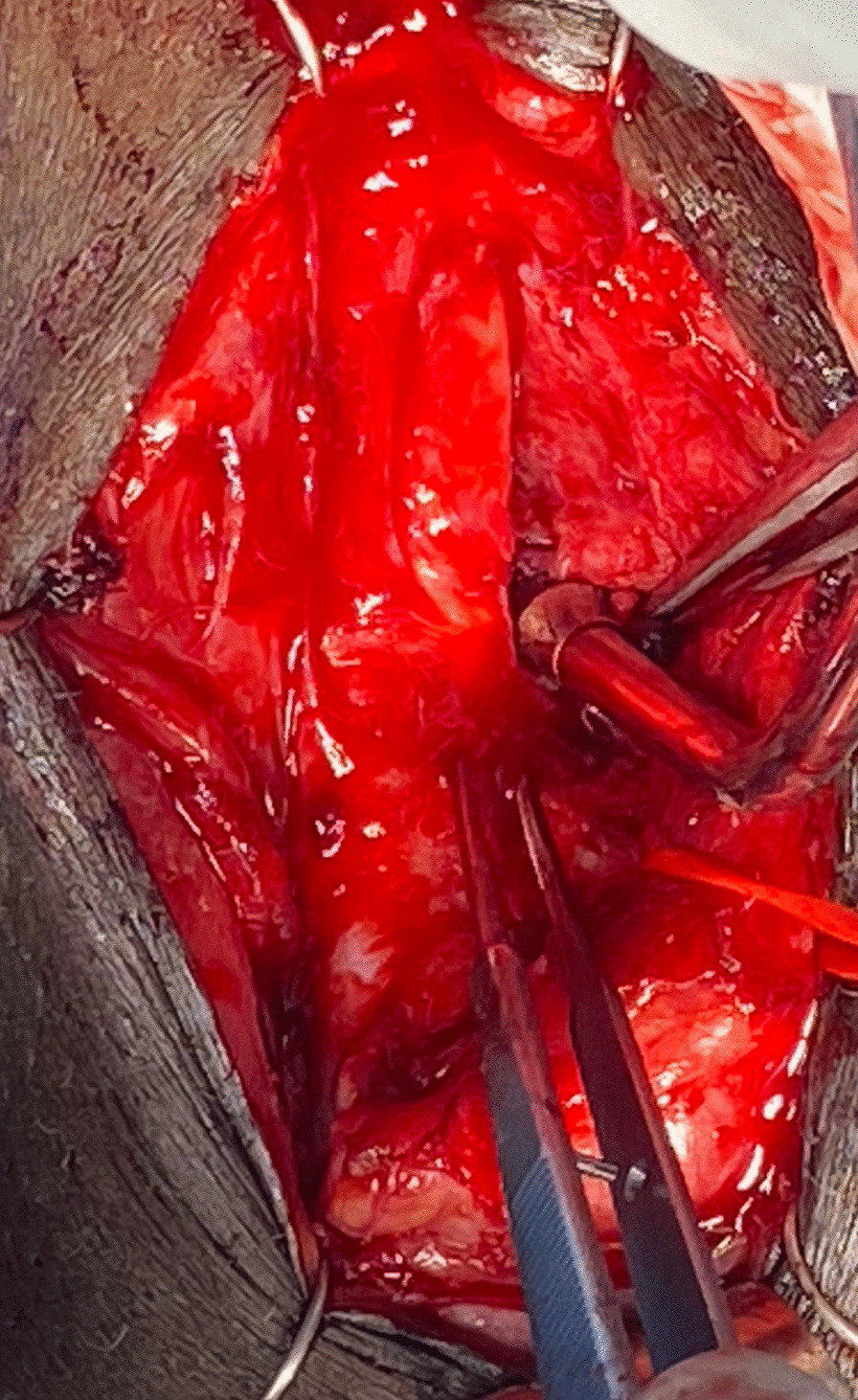
Urethrocavernous fistula. Tip of suction tip denotes fistulous tract between the bulbar urethra and the left corporal body (at time of second surgery with our team)

**Fig. 4:**
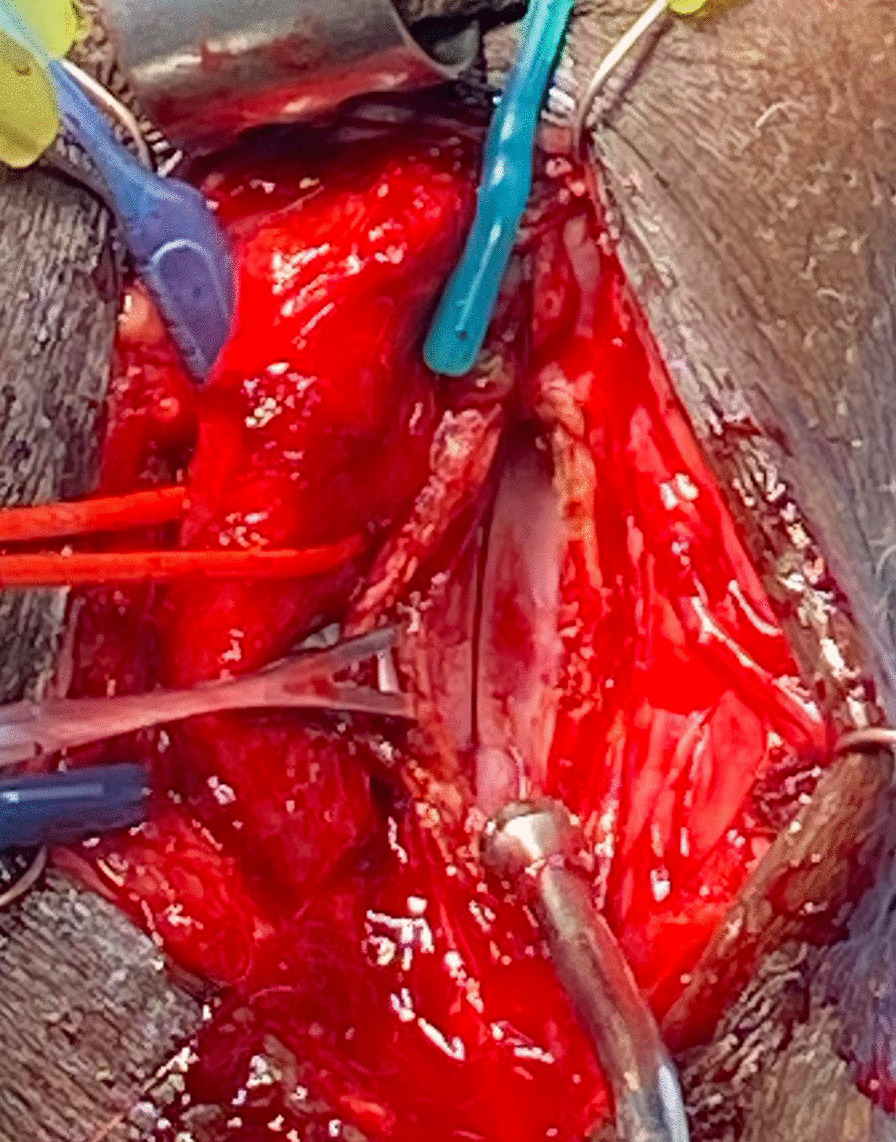
Urethrocavernous fistula. Wire used to cannulate urethral fistula is noted entering left corporal body (as seen after left corporotomy is made) and left malleable implant has been removed (at time of second surgery with our team)

Intraoperative urethroscopy showed a pinpoint opening in the left bulbar urethra concerning for fistulous tract which was able to be cannulated with a wire (Fig. 3). Perineal exploration confirmed the wire exiting the bulbar urethra and entering the adjacent left corporal body (Fig. 4). The fistulous tract was excised and a non-transecting bulbar urethroplasty was performed. Two-week post-operative peri-catheter RUG showed no evidence of contrast extravasation and the catheter was removed. There was no evidence of recurrent scrotal abscess on exam. Despite multiple complications, the patient remains interested in future IPP placement.

## Discussion and conclusions

This case illustrates multiple rare complications of penile prosthesis placement: bulbar urethral IPP cylinder erosion and secondary contralateral urethrocavernous fistula development.

The overall rate of reoperation for penile prosthesis procedures approaches 15 % after 10 years but is highest in the first year after surgery [8]. African-American, Hispanic, and younger men have a higher risk of re-operation. In regards to his first complication, device erosion more commonly involves the distal penile urethra. Rates of erosion range from 1 to 11 % and are more common in redo cases and in those with corporal fibrosis [3]. Diabetic patients, including the patient reported here, are at higher risk of corporal fibrosis.

Urethral injury is a rare complication of IPP placement. When recognized intraoperatively, the injury should be repaired and prosthetic placement aborted. Unrecognized urethral injury can present months after surgery with variable symptoms, including prosthetic infection, and is more commonly associated with MPP than IPP. Though the standard of care in suspected intraoperative urethral injury is repair of urethral injury and aborting prosthesis placement, some have reported placing a prosthetic cylinder in the non-injured side [2]. Some have even performed suprapubic cystotomy to proceed with prosthesis placement after a urethral injury is detected [9] but these reports are rare.

One unique aspect of this case is that the complications occurred proximally in the bulbar urethra. Urethral erosion is more likely to occur at the distal penis near the glans, where the two cavernosal bodies converge, and the prosthetic cylinders create pressure on the distal penile urethra [2]. This further suggests that our patient sustained an unrecognized proximal corporal and/or bulbar urethral injury during corporal dilation, or less likely corporotomy at the time of his redo IPP placement leading to device erosion into the bulbar urethra. Other possibilities include peri-operative infection resulting in erosion or less likely, friction from simply using the device as directed.

Given that his second urethral complication involved the contralateral corpora, we chose to refer to this as a secondary, de novo urethrocavernous fistula since failure of the initial repair would present ipsilaterally. Our patient required extended 2-week catheter drainage for healing of his initial urethroplasty at the time of unilateral MPP placement. We suspect the pressure from MPP while the catheter remained in place contributed to poor wound healing and eventual secondary contralateral fistula formation. His risk of poor healing was further elevated by poorly controlled diabetes. This fistula led to persistent urinary extravasation causing recurrent scrotal abscesses and glanular pain even though the fistula was too small to identify on office cystoscopy.

Urethrocavernous fistula is a rare complication of penile prosthesis placement. Urethral erosion and/or fistula has been reported in up to 80 % of patients with chronic indwelling or intermittent catheterization secondary to friction and inflammation [10]. Though our patient was not catheter dependent, he did require a catheter for postoperative urethral healing which likely increased his risk for fistula formation. We intentionally chose a small-bore (14 French), catheter as a larger catheter would increase pressure on the urethral repair, and possibly increase the risk of this complication. Given the risk of fistula formation in catheter-dependent patients, some have reported performing perineal urethrostomy or suprapubic cystotomy at the time of penile prosthesis surgery [11]. Caraceni et al. reported a case of urethrocavernous fistula with successful IPP replacement, primary repair of urethral and cavernosal defects augmented by the use of a dry layer of the human coagulation factors fibrinogen and thrombin [12]. Given the paucity of cases of urethral erosion and urethrocavernous fistula secondary to penile prosthesis, no standard of care has been established.

Urethral erosion and urethrocavernous fistula formation are rare complications of penile prosthesis placement. It is important understand that patients with corporal fibrosis, diabetes, redo prosthesis surgeries, and those requiring catheterization will be at elevated risk of recurrent and de novo urethral complications. Presenting symptoms of these complications vary (in this case, only symptom was persistent urethral discharge) and may require careful consideration to successfully diagnose and repair.

## Data Availability

Not applicable.

## Abbreviations

IPP: Inflatable penile prosthetic
MPP: Malleable penile prostheses
EPA: Excision and primary anastomosis
RUG: Retrograde urethrogram
